# Perineural pretreatment of bee venom attenuated the development of allodynia in the spinal nerve ligation injured neuropathic pain model; an experimental study

**DOI:** 10.1186/1472-6882-14-431

**Published:** 2014-11-04

**Authors:** Won Uk Koh, Seong Soo Choi, Jong Hyuk Lee, So Hee Lee, Sun Kyung Lee, Yoon Kyung Lee, Jeong Gil Leem, Jun Gol Song, Jin Woo Shin

**Affiliations:** Department of Anesthesiology and Pain Medicine, Asan Medical Center, University of Ulsan, College of Medicine, Pungnap-2Dong, Songpa-Gu, Seoul 388-1 Korea; Department of Anesthesiology and Pain Medicine, Hangang Sacred Heart Hospital, Hallym University Medical Center, Youngdeungpo-Dong, Youngdeungpo-Gu, Seoul, Korea

**Keywords:** Allodynia, Bee venom, Neuropathic pain, Transient receptor potential

## Abstract

**Background:**

Diluted bee venom (BV) is known to have anti-nociceptive and anti-inflammatory effects. We therefore assessed whether perineural bee venom pretreatment could attenuate the development of neuropathic pain in the spinal nerve ligation injured animal model.

**Methods:**

Neuropathic pain was surgically induced in 30 male Sprague Dawley rats by ligation of the L5 and L6 spinal nerves, with 10 rats each treated with saline and 0.05 and 0.1 mg BV. Behavioral testing for mechanical, cold, and thermal allodynia was conducted on postoperative days 3 to 29. Three rats in each group and 9 sham operated rats were sacrificed on day 9, and the expression of transient receptor potential vanilloid type 1 (TRPV1), ankyrin type 1 (TRPA1), and melastatin type 8 (TRPM8) receptors in the ipsilateral L5 dorsal root ganglion was analyzed.

**Results:**

The perineural administration of BV to the spinal nerves attenuated the development of mechanical, thermal, and cold allodynia, and the BV pretreatment reduced the expression of TRPV1, TRPA1, TRPM8 and c − Fos in the ipsilateral dorsal root ganglion.

**Conclusion:**

The current study demonstrates that the perineural pretreatment with diluted bee venom before the induction of spinal nerve ligation significantly suppresses the development of neuropathic pain. Furthermore, this bee venom induced suppression was strongly related with the involvement of transient receptor potential family members.

## Background

Traditionally, bee venom (BV) has been utilized in alternative and complementary medicine to treat and relieve pain in patients with rheumatoid arthritis and osteoarthritis [1]. In oriental alternative medicine, the injection of BV into an acupuncture point, a practice called apipuncture, has been believed to relieve pain and inflammation, restoring normal body functions [1, 2]. By contrast, the BV has been found to be a potent nociceptor that causes pain and inflammation, as well as causing potent allergic reactions in sensitized individuals [3–6].

The nociceptive and inflammatory effects of BV have made it useful in developing animal models of inflammatory pain. Since its introduction [4], the BV pain test has been used widely in experimental studies [3]. For example, subcutaneous injection of BV into rat hind paws induces inflammation in that paw, as well as primary thermal and mechanical hyperalgesia, secondary thermal hyperalgesia, and mirror image heat hyperalgesia [3, 7].

Paradoxically, diluted BV (DBV) has been shown to have anti-nociceptive and anti-inflammatory properties. Most experimental animal studies with DBV have focused on its effects and mechanisms of action during injections into acupuncture points, mostly at the Zusanli point, an acupuncture point located in the hind limb [8–10]. Previous studies have revealed that the anti-nociceptive and anti-hyperalgesic effects of DBV are strongly associated with alpha2-adrenoreceptor activation and capsaicin insensitive primary afferent fibers, but not with opioid receptors [9, 11]. The anti-arthritic effects of DBV have been shown to involve the inactivation of nuclear factor kappa B, which inhibits the release of inflammatory cytokines [12], and an increase in endogenous glucocorticoid concentration [13]. The specificity of BV in acupoint injection is unclear, as the injection of chemical irritants other than BV into the Zusanli acupuncture point also produced significant anti-nociceptive effects [14]. Moreover, when the transient receptor potential (TRP) cation channel family was investigated, the involvement of capsaicin–sensitive and –insensitive afferents in the anti-nociceptive mechanism of BV yielded conflicting results [11, 14].

The mechanisms underlying the anti-allodynic effects of BV are unclear. Acupoint injection of DBV in animals with peripheral nerve injury was shown to increase the threshold against thermal and cold stimuli [9]. The sensitization of TRP receptor families has been found to play an important role in the development of cold and thermal allodynia [15–17].

In the present study, we tested the hypothesis that perineural DBV pretreatment may attenuate the development of neuropathic pain, and that DBV may desensitize TRP cation channel sensitive nociceptive afferents, including vanilloid type 1 (TRPV1), ankyrin type 1 (TRPA1), and melastatin type 8 (TRPM8) receptors, which may result in anti-nociceptive and anti-allodynic effects in the rat spinal nerve ligation (SNL) injury model of neuropathic pain.

## Methods

### Experimental animals

The study protocol was approved by the Institutional Animal Care and Use Committee (IACUC) of Asan Medical Center. Male Sprague Dawley rats (Orient Bio, Sungnam, Korea), weighing 180–200 g, were housed individually for 7 days in humidity and temperature controlled (21 ± 1°C) vivaria, with a 12-hour night/day cycle (07:00 hr onset) and free access to food and water at all times, before use in experiments. Behavioral testing and analgesiometry were performed according to the ethical guidelines set by the IACUC of Asan Medical Center, and the animals were euthanized after completion of planned tests.

### Experimental groups and drug preparation

Neuropathic pain was surgically induced in 30 male rats by ligation of the L5 and L6 spinal nerves [18], with 10 rats each treated with saline and 0.05 and 0.1 mg DBV. Three rats in each group were sacrificed 9 days postoperatively for immunohistochemical assay, and behavioral test results were obtained from seven rats in each group. Sham surgery without nerve ligation was also performed in 3 rats per experimental group (total 9 rats) for reference data.

Bee venom was extracted from *Apis mellifera* (Apitoxin®Guju Pharm., Seoul, Korea) and dissolved in 0.9% normal saline at concentrations of 1 μg/μL and 2 μg/μL. The experimenter was blinded to the composition of the experimental drug administered.

### The induction of neuropathic pain and DBV treatment

The animals were anesthetized by intraperitoneal injection of zoletil (12.5 mg) and xylazine (3 mg) and placed on the operating table. An approximately 1.5 cm midline incision was made on the left lateral side of each rat at the L5–L6 level. The paravertebral muscles were dissected and retracted, and the L5 and L6 vertebral bodies were partially exposed. The left L5 transverse process was partially excised and the left L5 and L6 spinal nerves were exposed. DBV solution or normal saline was injected into the perineural sheath of the L5 and L6 spinal nerves using a microinjection syringe. Approximately 5 mm of the exposed spinal nerves were bathed for 5 min with DBV solution or normal saline. The wound was closed with 4-0 black silk (AILEE, Busan, Korea) sutures and the animals were allowed to recover. One hour after full recovery, the animals were again anesthetized and the spinal nerves were again exposed as described above, and neuropathic pain was surgically induced by ligation of the L5 and L6 spinal nerves as previously described [18].

### Behavioral assessment

To eliminate the effect of errors associated with diurnal rhythm, all behavioral tests were performed at the same time of the day. Behavioral testing for mechanical, cold, and thermal allodynia was performed, starting from postoperative day 3, on the injured ipsilateral hindpaw.

For the mechanical and cold allodynia tests, the rats were placed in individual acrylic cages with a wire mesh floor and were allowed to habituate for 30 min. The tactile threshold for mechanical allodynia was measured by applying eight calibrated von Frey filaments (0.41–15.1 g; Stoelting Co., Wood Dale, IL, USA) to the palm of the injured ipsilateral hind paw. Sufficient pressure was applied for 6 seconds to cause slight bending of the filament against the midplantar surface of the injured hind paw, with a brisk withdrawal or flinching of the paw considered a positive response. The 50% withdrawal threshold was determined by using the up-down method [19]. The cut-off value was defined as the absence of response to 15.1 g force.

Cold allodynia against chemical stimuli was tested using the acetone drop application technique [20]. Briefly, a drop of acetone was applied five times, at intervals of 3 minutes, to the midplantar surface of the injured ipsilateral hind paw using a syringe connected to a thin polyethylene tube, and the frequency of paw withdrawals in response to acetone was recorded.

Thermal allodynia and cold allodynia against heat was analyzed using a hot/cold plate apparatus (UgoBasile, Comerico, Italy) [21]. For thermal allodynia testing, each rat was placed on the hot plate device, with the temperature adjusted to 42 ± 0.1°C. For cold allodynia testing, the same device was used with the temperature adjusted to 10 ± 0.1°C. The latency to first nocifensive behavior (hind paw withdrawal or liking) was regarded as an index to nociceptive threshold. Each rat was tested three times with sufficient intervals between trials to avoid possible effects of anesthesia or tissue damage. The cut-off time was set at 30 seconds for thermal and 100 seconds for cold allodynia to prevent possible tissue damage to the affected paw.

Baseline values were determined by performing these behavioral tests 1 day prior to neuropathic surgery. Behavior was also measured at 3, 5, 7, 9, 13, 17, 21, 25, and 29 days after neuropathic pain surgery and drug administration. The mechanical withdrawal threshold was reported as actual threshold in grams (g); the withdrawal frequency in response to cold stimuli per rat was reported as percentage (%), by dividing the number of paw withdrawals by five and multiplying the result by 100; and the withdrawal latency against heat and cold was reported as elapsed time (s) until the start of the withdrawal response.

### Immunohistochemistry

Three rats in each group and 9 sham operated rats were sacrificed on postoperative day 9. The animals were anesthetized by intraperitoneal injection of zoletil (12.5 mg) and xylazine (3 mg), and fixative containing 4% buffered paraformaldehyde was perfused through the left ventricle. The left L5 DRG from the rats were dissected and fixed immediately in the same solution. Tissue samples were embedded in paraffin, and the blocked sections were cut (10 μm) with a microtome and then mounted onto slides. These samples were deparaffinized with three changes of xylene for 5 minutes each, and hydrated in 100% and 95% ethanol for 10 minutes each and in 70% ethanol for 5 minutes, and rinsed twice in distilled water for 5 minutes each. The slides were immersed in 0.01 M sodium citrate buffer (pH 6.0), which was heated at 95°C for 15 minutes. The immersed slides were then allowed to cool in air for 30 minutes. The sections were blocked with 5% normal donkey serum, 0.3% Triton X-100 and 1% bovine serum albumin (BSA) in phosphate buffered saline–Tween (PBS-T) for 1 hour.

The sections were subsequently incubated overnight at 4°C with rabbit polyclonal antibodies to TRPV1 (1:1000; ab31895, Abcam, Cambridge, UK), TRPA1 (1:1000; ab68847, Abcam, Cambridge, UK), or TRPM8 (1:1000; ab104569, Abcam, Cambridge, UK); with the IgG fraction of mouse polyclonal antibody to neurofilament 200KDa (NF200), a marker of myelinated neurons (1:1000; n0142, Sigma, St Louis, MO); or with the IgG fraction of sheep polyclonal antibody to c–Fos, an indicator of neuronal activity (1:100; LS–C93966, LSBio, Seattle, WA). Sections incubated with antibodies to TRPV1, TRPA1, and TRPM8 \were subsequently incubated with Alexa Flour 546 donkey anti-rabbit IgG (red; 1:1000; Invitrogen, Carlsbad, CA) for 2 hours at room temperature. Sections incubated with antibodies to NF 200 and c-fos were subsequently incubated with Alexa Flour 488 donkey anti-mouse IgG (green; 1:1000; Invitrogen, Carlsbad, CA) and Alexa Flour 350 donkey anti-sheep IgG (blue; 1:1000; Invitrogen, Carlsbad, CA), respectively. The sections were rinsed in PBS-T and mounted onto slides, and immunofluorescence was assayed using a confocal fluorescent microscope (Olympus BX51 system microscope, Tokyo, Japan) with imaging software (Image Pro Plus ver. 5.1, Media Cybernetics, Rockville, MD, USA).

The number of immunoreactive (IR) cells was counted in six slices of the DRG for each group. The percentages of IR cells, doubly and triply labeled cells were calculated by dividing the observed numbers of these cells by the total number of cells observed in the field and multiplying by 100.

### Statistical analysis

All data are presented as means ± standard errors (SEM). Behavioral results were compared in the experimental and control groups by two-way repeated measure of analysis of variance (ANOVA) followed by post–hoc analysis using the Holm–Sidak test. The percentages and numbers of TRPV1, TRPA1, and TRPM8 positive cells in the DRG were compared using the Kruskal–Wallis test or the chi-square test, as appropriate. SigmaPlot version 12.0 (Systat Software Inc, Richmond, CA, USA) was used for data analysis. P < 0.05 was considered statistically significant.

## Results

### The results of behavioral testing after DBV treatment

**Figure 1:**
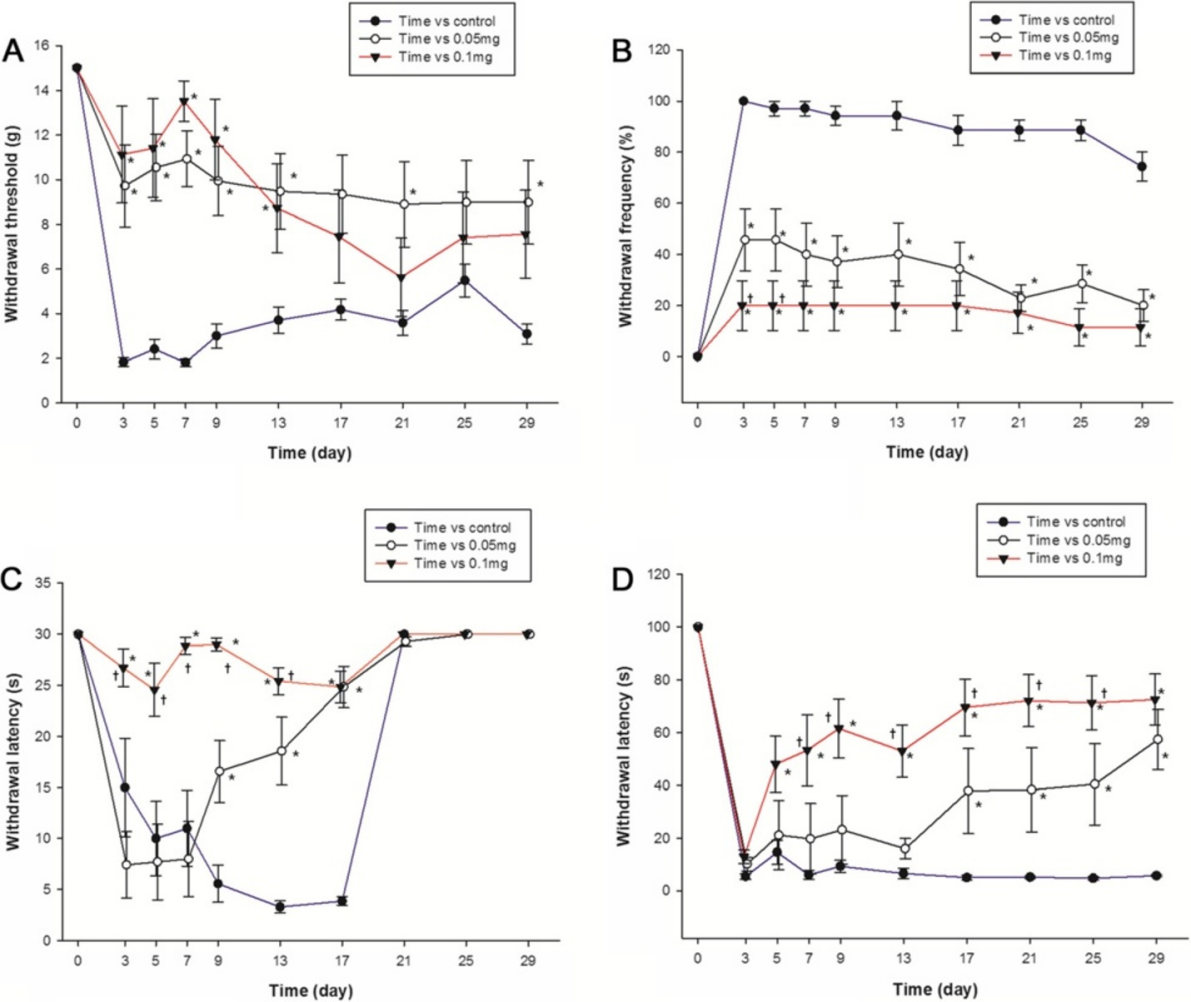
**The results of behavioral testing after perineural diluted bee venom (DBV) pretreatment in time–course. A**. Mechanical allodynia was suppressed in rats pretreated with diluted bee venom (DBV), relative to the control group. The paw withdrawal threshold in both DBV treated groups were significantly higher compared with the control group from observation day 3 to day 13. **B**. DBV effect on acetone stimulation test in rats. Cold allodynia was inhibited, beginning on day 3 in both groups of rats treated with DBV, compared with the control group and continued until the end of study. **C**. DBV effect on hot plate test in rats. Thermal allodynia was inhibited from day 3 until day 17 in 0.1 mg DBV group, and day 9 to day 17 in 0.05 mg DBV group. The PWL of control and both BV treated groups restored to normal values beginning on day 21. The preservation of PWL was dose related, being significantly higher in rats treated with 0.1 mg than with 0.05 mg DBV on days 3, 5, 7, 9, and 13. **D**. DBV effect on cold plate test in rats. The development of cold allodynia after SNL was inhibited in the rats pretreated with DBV, especially in the 0.1 mg group, beginning on day 5 until the end of study. The 0.1 mg DBV group presented significantly longer PWL compared with 0.05 mg group throughout the study period. Each point represents mean ± standard error of the mean (SEM). *p < 0.05 compared with the control group. †*p* < 0.05 compared with the BV 0.05 mg group. n = 7 animal per group.

The perineural pretreatment of the L5 and L6 spinal nerves with DBV significantly attenuated the development of mechanical allodynia. The mechanical hind paw withdrawal threshold (PWT) was significantly higher in rats treated with 0.05 mg and 0.1 mg DBV on days 3, 5, 7, 9 and 13 compared with the saline treated control group animals (Figure 1). Although surgically treated control animals developed significant cold allodynia against acetone stimulation, rats perineurally treated with DBV showed inhibition of cold allodynia development, producing significantly lower paw withdrawal frequency (PWF). This inhibition of cold allodynia in the both DBV treated groups was observed on the first day of behavioral testing and continued until the end of the study, lasting more than 4 weeks (Figure 1). Perineurally administered DBV also significantly inhibited the development of both thermal and cold allodynia against hot/cold plate test, producing a significantly higher threshold in hind paw withdrawal latency (PWL) than control rats (Figure 1). This inhibition against temperature stimulation presented to be dose related. Significant greater inhibition of thermal and cold allodynia was observed in rats treated with 0.1 mg than with 0.05 mg group DBV. Beginning on day 21, however, response to thermal stimuli was diminished in both DBV treated and control groups, with the PWL restored to preoperative values. In contrast, the response to cold plate test was inhibited until the end of the study.

### Immunohistochemical analysis of the DRG

**Figure 2:**
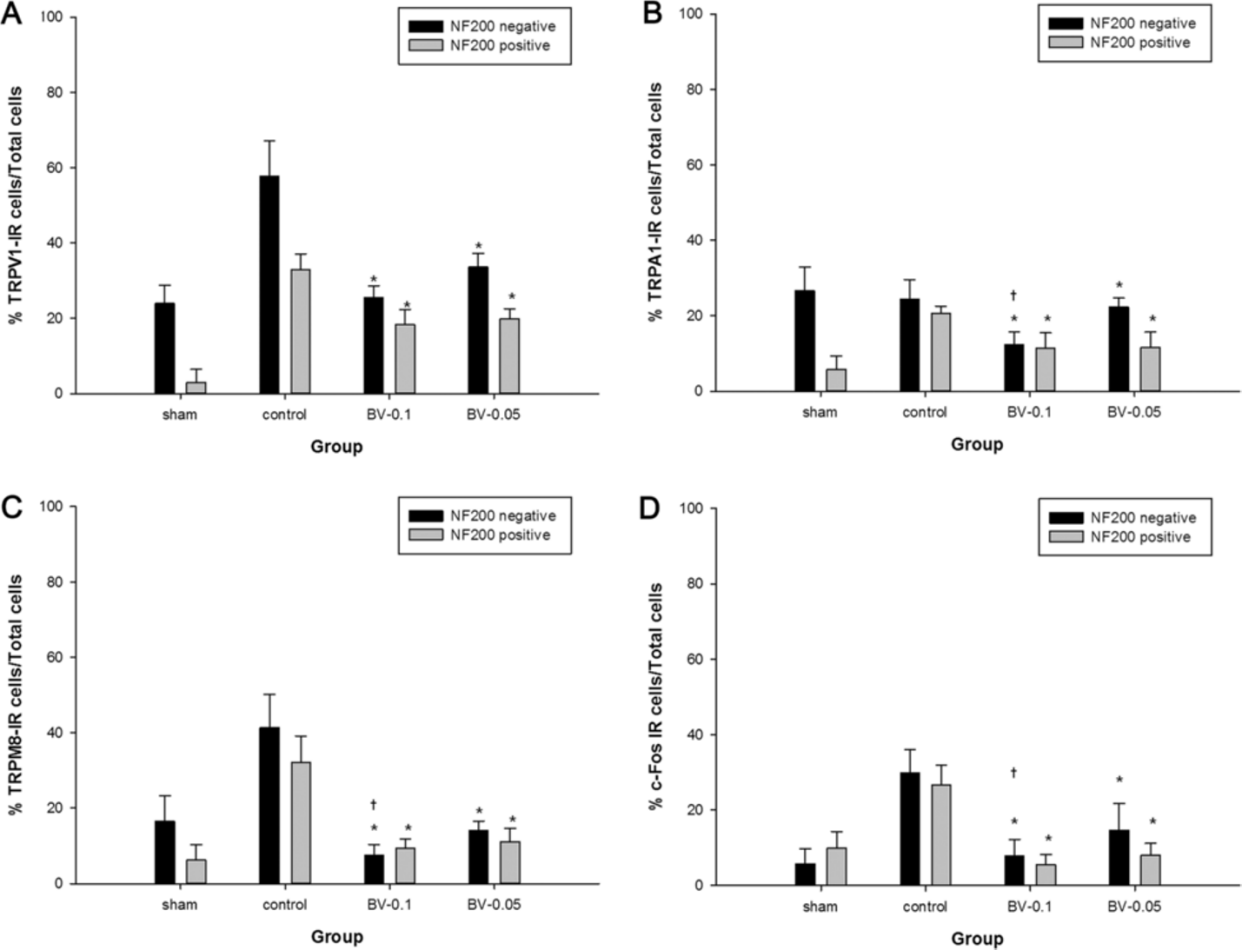
**The proportion of immunoreactive (IR) neurons observed at the ipsilateral L5 dorsal root ganglion (DRG).** Histograms show the proportion of **A**. transient receptor potential vanilloid type 1 (TRPV1), **B**. ankyrin type 1 (TRPA1), **C**. melastatin type 8 (TRPM8) and **D**. c–Fos IR neurons. Compared with sham operated animals, spinal nerve ligation surgery (SNL) significantly increased the proportion of neurofilament (NF) 200 positive neurons presenting immunoreactivity for TRPV1, TRPA1, TRPM8 and c–Fos. In the NF200 negative neurons, SNL surgery significantly increased the proportion of TRPV1, TRPM8 and c–Fos IR neurons in the dorsal root ganglion. Perineural pretreatment with 0.05 mg or 0.1 mg diluted bee venom (DBV) significantly decreased the proportion of NF 200 positive and negative neurons expressing TRPV1, TRPA1, TRPM8 and c–Fos compared with the control group. Three rats in each group were analyzed. Each bar represents mean (%) ± standard deviation (SD). *p < 0.05 compared with the control group, † *p* < 0.05 compared with the BV 0.05 mg group.

**Figure 3:**
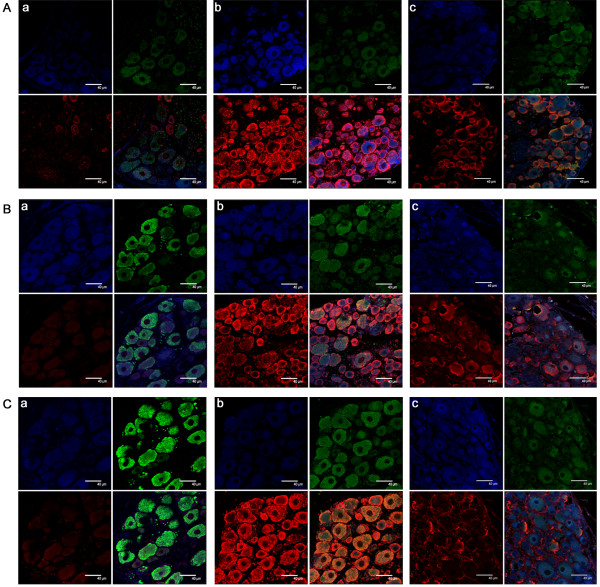
**Immunofluorescent images of dorsal root ganglion. A**. Transient receptor potential vanilloid type 1 (TRPV1) immunoreactive (IR) neurons and merged images with neurofilament (NF) 200 and c–Fos observed through a confocal microscope. Immunofluorescent images showing neurons positive for TRPV1 (red), NF200 (green) and c–Fos (blue) in the dorsal root ganglion (DRG) 9 days after surgery in a) sham operated, b) spinal nerve ligation (SNL), and c) 0.1 mg diluted bee venom (DBV) treated SNL rats. Clockwise from upper left: c–Fos– NF200 –merged – TRPV1. **B**. Transient receptor potential ankyrin type 1 (TRPA1) IR neurons and merged images with NF200 and c–Fos. Neurons positive for TRPA1 (red), NF200 (green) and c–Fos (blue) in the DRG 9 days after surgery in a) sham operated, b) SNL, and c) 0.1 mg DBV treated SNL rats. Clockwise from upper left: c–Fos– NF200 –merged – TRPA1. **C**. Transient receptor potential melastatin type 8 (TRPM8) IR neurons and merged images with NF200 and c–Fos observed through a confocal microscope. Neurons positive for TRPM8 (red), NF200 (green) and c–Fos (blue) in the DRG 9 days after surgery in a) sham operated, b) SNL, and c) 0.1 mg DBV treated SNL rats. Clockwise from upper left: c–Fos– NF200 –merged – TRPA1. All images are magnified × 630, with scale bars = 40 μm. Three rats in each group were analyzed.

Compared with the sham operated group, the surgically treated control group showed a significant increase in the proportion of TRPV1 expressing neurons in the DRG (p < 0.05). The proportion of TRPV1–IR neurons in the DRG of the both DBV treated group was significantly reduced compared with the control group (Figures 2 and 3). SNL surgery resulted in a significant increase in TRPA1–IR cells in NF200 negative unmyelinated neurons compared with the sham operated group (p < 0.05). DBV pretreatment significantly reduced the numbers of TRPA1–IR cells in both myelinated and unmyelinated neurons when compared with the control group. In the unmyelinated neurons, greater reduction of TRPA1–IR cells was observed in the BV 0.1 mg group compared with the BV 0.05 mg group, but a similar dose relationship was not observed in myelinated neurons (Figures 2 and 3). SNL surgery also induced a significant increase in the number of TRPM8–IR cells in both myelinated and unmyelinated neurons (p < 0.05), and DBV pretreatment suppressed the expression of TRPM8 in the DRG of SNL animals. Similarly with TRPA1 expression, a greater reduction in the number of unmyelinated TRPM8–IR neurons were observed in the BV 0.1 mg group compared with the 0.05 mg group (Figures 2 and 3).

The SNL surgery resulted in robust increases in c–Fos immunoreactivity (Fos–IR) in both myelinated and unmyelinated neurons (p < 0.05, Figures 2 and 3). DBV pretreatment significantly reduced the number of Fos–IR neurons in the DRG compared with the control group and in the unmyelinated neurons, the suppression of c–Fos activity was greater in the BV 0.1 mg group compared with BV 0.05 mg.

## Discussion

We examined the ability of perineurally administered DBV to inhibit the development of neuropathic pain in an animal model. Perineural pretreatment with DBV demonstrated inhibition in the development of mechanical, cold and thermal allodynia.

Several studies have examined the effects of DBV on neuropathic pain. DBV injection into the acupuncture point (Zusanli acupoint) resulted in a significant decrease in pain sensitivity and inflammatory reactions, as well as decreasing neuronal immunoreactivity in the spinal cords of animal pain model [8, 9]. A single subcutaneous injection of DBV into the Zusanli acupoint was shown to effectively decrease responses to thermal and cold stimuli in neuropathic animals, but did not attenuate mechanical allodynia [9, 10, 22]. Furthermore, repeated subcutaneous injections of DBV into the acupoint alleviated mechanical allodynia in the chronic constriction injury (CCI) model of neuropathic pain [23]. The behavioral results in our study were similar with these previous findings.

The mechanism of these anti–nociceptive effects of DBV is not clear and under investigation. The spinal alpha-2 adrenergic pathway has been shown to be important for the anti-nociceptive effects of DBV acupuncture in formalin induced inflammatory pain and CCI neuropathic pain models [9, 10, 22, 23]. By contrast, opioid receptors were not involved in the anti-nociceptive mechanism of DBV injection, whereas the involvement of capsaicin sensitive afferents is unclear [3, 14]. One study found that the effects of BV acupuncture were mediated through capsaicin insensitive primary afferents [11], whereas another study suggested that capsaicin sensitive primary afferent fibers are partially involved in the anti-nociceptive effects of DBV treatment [14]. We found that DBV significantly reduced the proportion of TRPV1–IR neurons at the DRG in the SNL model of neuropathic pain, suggesting that DBV pretreatment have down–regulated the activity of capsaicin sensitive afferent neurons. This down–regulation may be one of the mechanisms underlying the anti-nociceptive effects of DBV, especially in maintaining thermal threshold.

The development of thermal hyperalgesia following subcutaneous BV injection was highly associated with increased TRPV1 expression in the spinal dorsal horn, making BV a useful agent for inflammatory pain modeling [24–27]. The reasons for these contradictory activities of BV on TRPV1 receptors are currently unknown, but may be due to different locations and doses of BV injections. The BV is composed of numerous bioactive compounds, which may have different effects depending on the site of application, even at the same dose [8, 9, 14, 23]. We injected rats with 0.05 mg or 0.1 mg BV dissolved in 50 μL of normal saline, doses chosen in reference to effective anti–nociceptive doses [8, 9]. The dose required for a robust and prolonged pain response in rats is known to be ≥ 0.2 mg, although lower doses, under 0.05 mg, induced a short, mild pain response [14].

DBV injection was recently reported to significantly reduce cold allodynia in the CCI neuropathic pain model [22]. Our results are consistent with these findings, as BV pretreatment significantly suppressed the development of cold allodynia. We also assessed the involvement of TRPA1 and TRPM8 receptors in the reduction of pain behaviors in response to cold stimuli. Both TRPA1 and TRPM8 expressions are increased following peripheral nerve injury and mRNAs encoding both were significantly increased after CCI surgery and were correlated with the onset of cold allodynia [28]. The TRPA1 receptor is important in the development of cold and mechanical allodynia in inflammatory and neuropathic pain models [29–32]. Increased expression of TRPA1 in A-δ fibers plays a major role in the development of cold hypersensitivity in the SNL injury model [33]. Our results were consistent with these findings, as we observed no increase in the proportion of unmyelinated NF200 negative TRPA-IR neurons, but a significant increase in myelinated NF200 positive TRPA-IR neurons after SNL in the DRG. We also found that BV pretreatment down–regulated the expression of TRPA1 receptors in both myelinated and unmyelinated fibers, which seemed to have resulted in the attenuation of cold allodynia. The TRPM8 receptor is also involved in the development of cold allodynia, and the decreased expression of TRPM8 receptors after intrathecal application of TRPM8 antisense oligonucleotide resulted in decreased sensitivity to cold stimuli in the CCI model [34]. However, discordant results on the involvement of TRPM8 in cold allodynia have been reported. In the SNL model, TRPM8 was a minor component while and TRPA1 was the major component in the development of cold nociception [31]. In our observation, TRPM8 expression was significantly increased at the ipsilateral L5 DRG in both myelinated and unmyelinated neurons after SNL injury, along with increased responses to cold and mechanical stimuli. The expression of TRPM8 receptors was significantly reduced after DBV pretreatment, indicating that both TRPA1 and TRPM8 are involved in the development of cold allodynia in the SNL injury model.

For immunohitochemical analysis, we chose day 9 because the peak response to stimuli in the SNL neuropathic model occurs between days 5 and 14 [18]. Since the effects of DBV on mechanical allodynia was shorter lived than its effects on cold or thermal allodynia, the relative proportions of TRPV1, TRPA1 and TRPM8-IR neurons may have varied over time, and the result of cell counting may have changed if the immunohistochemical assay was performed at a different period.

## Conclusion

In conclusion, the perineural pretreatment of DBV effectively inhibited the development of neuropathic pain in the SNL animal model. DBV induced long-lasting inhibition of mechanical, thermal and cold allodynia. DBV-induced anti-nociception was apparently due to its inhibition of TRPV1, TRPA1, and TRPM8 receptors. Investigation of BV components may help in developing therapeutic modalities to treat neuropathic pain.

## Abbreviations

BSA: Bovine serum albumin
BV: Bee venom
CCI: Chronic constriction injury
DBV: Diluted bee venom
DRG: Dorsal root ganglion
IR: Immunoreactive
NF200: Neurofilament 200KDa
PWF: Paw withdrawal frequency
PWL: Paw withdrawal latency
PWT: Paw withdrawal threshold
PBS-T: Phosphate buffered saline-Tween
SNL: Spinal nerve ligation
TRP: Transient receptor potential
TRPA1: Transient receptor potential ankyrin type 1
TRPM8: Transient receptor potential melastatin type 8
TRPV1: Transient receptor potential vanilloid type 1.
